# Clinical trial network for the promotion of clinical research for rare diseases in Japan: muscular dystrophy clinical trial network

**DOI:** 10.1186/s12913-016-1477-4

**Published:** 2016-07-11

**Authors:** Reiko Shimizu, Katsuhisa Ogata, Akemi Tamaura, En Kimura, Maki Ohata, Eri Takeshita, Harumasa Nakamura, Shin’ichi Takeda, Hirofumi Komaki

**Affiliations:** Translational Medical Center, National Center of Neurology and Psychiatry, Tokyo, Japan; Institute of Clinical Research, National Hospital Organization Higashi-Saitama Hospital, Saitama, Japan; Department of Clinical Research Promotion, Center hospital, National Center of Neurology and Psychiatry, Tokyo, Japan; Department of Child Neurology, National Center of Neurology and Psychiatry, Tokyo, Japan

**Keywords:** Muscular dystrophy clinical trial network (MDCTN), Orphan drugs, Neuromuscular diseases, Rare diseases, Clinical trial network, Muscular dystrophy, Registry of Muscular Dystrophy (Remudy)

## Abstract

**Background:**

Duchenne muscular dystrophy (DMD) is the most commonly inherited neuromuscular disease. Therapeutic agents for the treatment of rare disease, namely “orphan drugs”, have recently drawn the attention of researchers and pharmaceutical companies. To ensure the successful conduction of clinical trials to evaluate novel treatments for patients with rare diseases, an appropriate infrastructure is needed. One of the effective solutions for the lack of infrastructure is to establish a network of rare diseases.

**Methods:**

To accomplish the conduction of clinical trials in Japan, the Muscular dystrophy clinical trial network (MDCTN) was established by the clinical research group for muscular dystrophy, including the National Center of Neurology and Psychiatry, as well as national and university hospitals, all which have a long-standing history of research cooperation.

**Results:**

Thirty-one medical institutions (17 national hospital organizations, 10 university hospitals, 1 national center, 2 public hospitals, and 1 private hospital) belong to this network and collaborate to facilitate clinical trials. The Care and Treatment Site Registry (CTSR) calculates and reports the proportion of patients with neuromuscular diseases in the cooperating sites. In total, there are 5,589 patients with neuromuscular diseases in Japan and the proportion of patients with each disease is as follows: DMD, 29 %; myotonic dystrophy type 1, 23 %; limb girdle muscular dystrophy, 11 %; Becker muscular dystrophy, 10 %. We work jointly to share updated health care information and standardized evaluations of clinical outcomes as well. The collaboration with the patient registry (CTSR), allows the MDCTN to recruit DMD participants with specific mutations and conditions, in a remarkably short period of time.

**Conclusion:**

Counting with a network that operates at a national level is important to address the corresponding national issues. Thus, our network will be able to contribute with international research activity, which can lead to an improvement of neuromuscular disease treatment in Japan.

## Background

Duchenne muscular dystrophy (DMD) is the most commonly inherited neuromuscular disease, affecting approximately 1 out of 3600 to 6000 live newborn boys. Many of the drugs and medical devices developed for the treatment of neuromuscular diseases are classified as orphan drugs [1]. In Japan, orphan drugs and medical devices are defined as “those intended for use in less than 50 000 patients, for which there is a high medical need.” Regarding therapeutic agents for rare diseases, orphan drugs have not been actively developed because they represent uncertain profit margins for pharmaceutical companies. However, this situation has changed drastically. Recently, various therapeutic approaches have been developed, and the conduction of clinical trials have either shown or are expected to show the efficacy of these therapeutic agents and strategies, such as antisense oligonucleotide drug and read-through therapy, for DMD treatment [2].

In conducting clinical research of rare diseases, patient recruitment, accurate evaluation and standardized treatment are important factors for assessing the effect of treatment. To recruit a sufficient number of patients, an international clinical research design is often chosen, and the appropriate infrastructure is needed to conduct this type of research.

Two well-known global networks have been organized to facilitate the study of novel therapies for neuromuscular diseases, including the TREAT-NMD in the European Union and the Cooperative International Neuromuscular Research Group (CINRG) in the United States. Each network has its own characteristic features, and is in regular and close communication with the other. The CINRG was established in 1999, as a consortium of medical and academic investigators from academic and research centers, to contribute with the improvement of therapeutic agents aimed for the treatment of neuromuscular diseases by conducting clinical observation studies and performing well-designed controlled clinical trials. Additionally, the coordinating center works as an academic research organization (ARO). Twenty-eight sites from 12 countries are collaborating for this purpose [3], and those sites are located at medical institutions employing specialists that care for patients with neuromuscular disease in each country. TREAT-NMD was established in 2007 as a network focused on the neuromuscular field that provides the infrastructure for conducting clinical research, such as patient registries, standardization of treatment, establishment of outcome measures, communication between patients, researchers, companies and regulation agencies for the purpose of ensuring that the most promising new therapies reach patients as quickly as possible. Members from 43 countries are gathered for this alliance [4, 5].

Japan has a long-standing history of treatment and care of patients with neuromuscular dystrophy. Since 1964, wards specifically designed to care for patients with neuromuscular dystrophy have been established in national hospitals, and these wards are managed by the Japanese government. This practice has gradually become widespread throughout Japan based on the government’s ruling. National hospitals were then reorganized in the National Hospital Organization (NHO) in 2004. The Ministry of Health, Labour and Welfare (MHLW) later designated the National Center of Neurology and Psychiatry (NCNP) to organize research groups for the treatment and medical care of patients with neuromuscular dystrophy in those hospitals. One of these research groups has performed annual surgeries and maintained an integrated database on neuromuscular dystrophy patients since 1999 [6]. This activity contributed to the improvement of treatment and care of patients with neuromuscular dystrophy, in particular, with the introduction of ventilators to the wards devoted to the care of these patients. Consequently, the mean age of death among patients with DMD in those specific wards was 17 years in 1980, 27.6 years in 2000 and 35.1 years in 2010. Ventilators were also distributed to the patients who were cared for at their own homes.

Muscular Dystrophy Clinical Trial Network (MDCTN) was established in 2012 as the result of activities of the clinical research group for neuromuscular dystrophy, including the NCNP and national hospitals, which have the specific wards and outpatient clinic to treat patients with neuromuscular dystrophy and the university hospitals. This is the first clinical research network for neuromuscular diseases in Asia, and is also the first clinical trial network for rare diseases in Japan aiming to expedite and ensure the success of clinical research for rare diseases.

## Methods

### Organizational structure

**Fig. 1:**
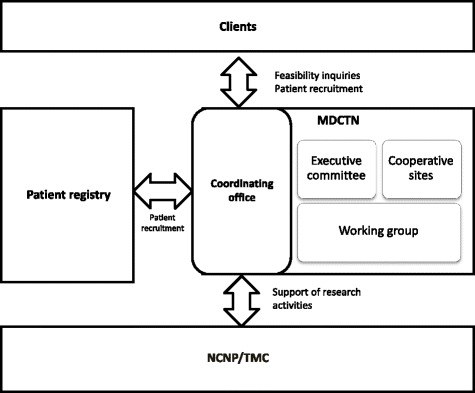
Structure of the Muscular dystrophy clinical trial network (MDCTN). This network consists of the steering committee, coordinating sites, working group and coordinating office. The coordinating office is the hub that communicates between clients, patient registry, and cooperative sites. The TMC supports research activities if necessary, and it provides protocol management. The MDCTN also includes a data manager, protocol development support, biostatistics analyses, and monitoring. Abbreviations: MDCTN, Muscular dystrophy clinical trial network; NCNP, National Center of Neurology and Psychiatry; TMC, Translational medical center

The structure of this network is shown in Fig. 1. The headquarters of this network are located at the Translational Medical Center (TMC), NCNP in Tokyo, Japan. The development of this network was supported by the Intramural Research Grant (23–6, 26–6) for Neurological and Psychiatric Disorders, provided by the NCNP. This network consists of 31 cooperative sites, and consists of a steering committee, specific working groups, and a coordinating office. The steering committee members include specialists in neuromuscular disease, 3 selected representatives from each cooperative sites, a director of the NCNP hospital, a director of the TMC at the NCNP, and a representative from the coordinating office. Any domestic medical institutions that are interested in clinical trials for neuromuscular diseases, and accept to complete the annual survey and non-disclosure agreement, are able to join in this network as cooperative sites. The coordinating office is the hub of this network, and collaborates with patient registries for the exchange of information. The coordinating office facilitates study participant recruitment. Additionally, it has been engaged in a consultation with clients and the coordination of processes related to the conduction of clinical research trials between researchers and pharmaceutical companies. The TMC will support these network activities and provide the experts, such as statisticians, project managers, data managers, and monitors, for these clinical research or trials, if necessary. The specific working groups are aimed at facilitating clinical research or trials. The outcome measure working group is now actively participating in a standardization of clinical outcome measures by verifying the outcome measures that can reflect the physical conditions of patients more accurately and ultimately evaluate the effect of interventions or therapies. This working group consists of medical doctors, physical therapists, and clinical coordinators, which have regular discussions aimed at looking for more sensitive and accurate outcome measures for multi-institutions by offering training courses, workshops and seminars for cooperative sites.

### Structure of the registry forms

Our site registry questionnaires are based on the Care and Treatment Site Registry (CTSR) of the TREAT-NMD [7], which will allow an easier collaboration between our network and TREAT-NMD in the future. In the list of the proportion of patient with each disease included in questionnaires for each coordination site, we added Fukuyama type neuromuscular dystrophy (FCMD), which is diagnosed relatively frequently in some Asian countries, such as Japan. We also added UDP-N-acetylglucosamine-2-epimerase/N-acetylmannosamine kinase (GNE) myopathy, and mitochondrial diseases to the original list proposed by TREAT-NMD. Conversely, hereditary motor neuropathy, congenital myasthenic syndrome, and muscular channelopathies have been excluded from our list because these diseases are rare in Japan and have not been included in the annual survey applied to the specific neuromuscular dystrophy wards in NHO. These questionnaires consist of the following items: basic information of each site, the total number of affected patients and number of patients diagnosed with each type of neuromuscular dystrophy, research activity, diagnostic techniques and equipment, clinical trial experience, and a list of available medical specialists for clinical trials. These questionnaires are annually sent and answers are collected online and manually curated by the coordinating office of the MDCTN.

### The management of patient recruitment

**Fig. 2:**
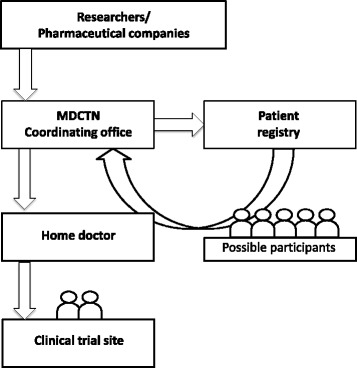
Patient recruitment pathway. First, the client (researcher or pharmaceutical company) requests the patient recruitment to Muscular dystrophy clinical trial network (MDCTN) coordinating office. The coordinating office communicates with the patient registry and sends recruitment letters to registered patients who might the meet criteria. Through communications with patients and their local doctor, participants are able to enter the clinical trial

Pharmaceutical industries or researchers contact the coordinating office of MDCTN to recruit participants into their clinical trials via collaboration with patient registries, such as the Registry of Muscular Dystrophy (Remudy) [8]. Then, the coordinating office will contact the patient registry office and ask to send recruitment letters to candidates according to their clinical and genetic data base. Candidates would send responses to the coordinating office, after being contacted as potential participants. Our coordinating office asks them to consult their doctors to confirm that they truly fit the inclusion and exclusion criteria of the proposed study. If they fulfill the criteria, the coordinating office introduces patients to the representatives of the institution where the clinical trial will be conducted (Fig. 2).

### The management of the feasibility inquiry

The operation of feasibility inquiries from pharmaceutical companies or researchers is one of the most important tasks of the coordinating center. Under the contract of confidentiality, the coordinating office collects the information regarding feasibility inquiries of their clinical trials or studies. Clients are able to choose the sites based on the information provided by the office.

### Regular meeting opportunities

For the exchange of opinions and information regarding updated research progress and current and promising therapies, we conduct an annual workshop with network members, representatives of pharmaceutical companies, patient advocacy groups, regulatory agencies, and anyone interested in MDCTN activities. We also have regular meetings with representatives from the cooperative sites to maintain a constant flow of communication among the sites. As a part of our working group activities, we conduct seminars for Physical Therapists (PT) and clinical research coordinators (CRCs) to standardize clinical evaluations.

### Ethics

This network does not have a central Institutional Review Board (IRB) system yet. The IRB of each cooperative site has to review the protocols for clinical research planned to be conducted at each institution. The data presented herein are not data, rather they consist of inquiries made to the cooperative sites.

## Results

### Cooperative sites

**Fig. 3:**
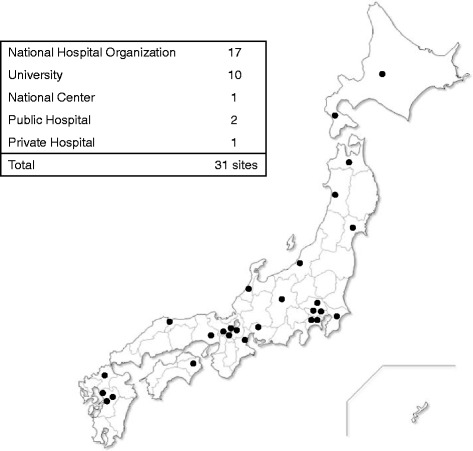
Map of the cooperative sites. Thirty-one sites have joined the Muscular dystrophy clinical trial network and are widespread across Japan. Several urban sites are concentrated in the large cities, such as Tokyo and Osaka. (Source http://www.mdctn.jp/network.html)

The MDCTN was established in December 2012, and a total of 31 institutions have registered since then: 17 national hospital organizations (NHO), 10 university hospitals, 1 national center, 2 public hospitals, and 1 private hospital. These institutions are distributed in almost all areas of Japan (Fig. 3).

One of the university hospitals organized their own clinical network for neuromuscular dystrophy in its district and registered the MDCTN as the representative of their network. Most of clinical departments who have registered as cooperative sites of MDCTN are either departments of neurology, pediatrics or pediatric neurology. One orthopedic department from a university hospital, characterized for the surgical treatment of scoliosis in patients with neuromuscular diseases, has also registered. One site did not submit their data in time, and thus, the data of that site were excluded. The data of the CTSR is an aggregate of data from 30 sites. Three of the cooperative sites have already registered in the CTSR of the TREAT-NMD.

### Description of site registry data

Thirty out of 31 cooperating sites answered the following queries between September 2013 and February 2014.

### Patient proportion

**Fig. 4:**
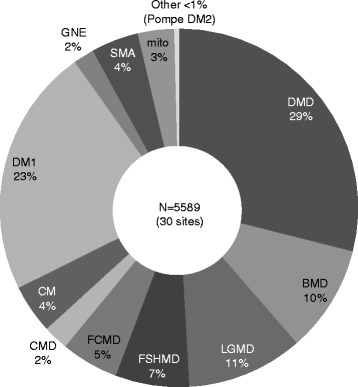
Overall patient cohort of the MDCTN. From 30 out of 31 cooperative sites, 5,589 patients have been reported. This data were calculated based on a rough estimate of the proportion of patients with each disease reported at each site. Abbreviations: MDCTN, Muscular dystrophy clinical trial network; GNE, UDP-N-acetylglucosamine-2-epimerase/N-acetylmannosamine kinase; DM1 and 2, myotonic dystrophy type 1 and type 2; DMD, Duchenne muscular dystrophy; LGMD, Limb-girdle muscular dystrophy; BMD, Becker muscular dystrophy; FCMD, Fukuyama type neuromuscular dystrophy; FSHMD, Facioscapulohumeral muscular dystrophy; CM, Congenital myopathy; CMD, Congenital muscular dystrophy; mito, Mitochondrial myopathy

**Fig. 5:**
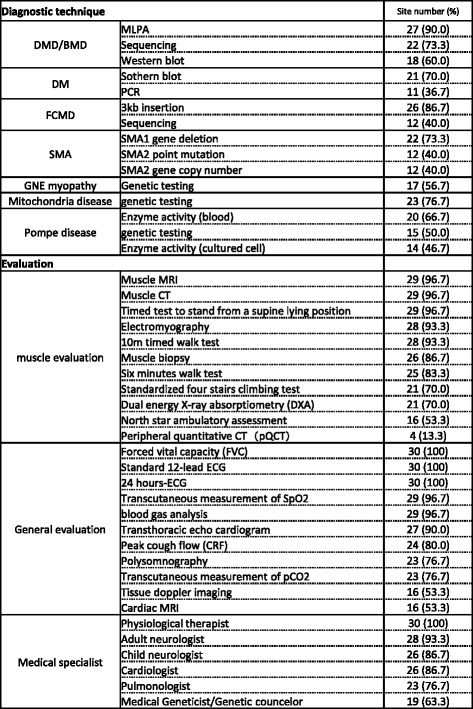
Available evaluations. This figure shows the number of sites, which provide the listed evaluations. This data were obtained from 30 out of 31 site reports

There is a total 5,589 patients with neuromuscular diseases (Fig. 4). In decreasing order, the diseases are listed as follows: DMD, 29 %; myotonic dystrophy type 1 (DM1), 23 %; Limb girdle muscular dystrophy (LGMD), 11 %; Becker muscular dystrophy (BMD), 10 %; facioscapulohumeral muscular dystrophy, 7 %; and FCMD, 5 %. Less than 1 % of patients registered have DM2 and Pompe disease. Approximately a half of DMD and two-thirds of BMD patients have reached adulthood (Data not shown). The current situation of each MDCTN sites, such as diagnostic techniques, clinical evaluation, medical specialist availability, and clinical trial experience, are described below and summarized in Fig. 5.

### Diagnostic techniques

The diagnosis for dystrophinopathies (DMD and BMD), such as multiplex ligation-dependent probe amplification analysis, sequencing, and genetic tests for DM and FCMD are covered by the national health insurance system in Japan. There are medical institutions, including the NCNP, which perform gene analysis for spinal muscular atrophy, mitochondrial myopathy, or GNE myopathy.

### Clinical evaluation

Timed tests, such as the 10-meter walking test, are largely available in all the sites. However, many sites have less experience in more complicated evaluations such as the North Star Ambulatory Assessment. Twenty-five cooperative sites can perform muscle biopsy. All sites can perform muscle magnetic resonance imaging (MRI) and one third of the sites can perform cardiac MRI as well.

### Medical specialist availability

Certified child neurologists are available in 19 cooperative sites, and certificated adult neurologists are available in 28 sites. Pulmonologist, cardiologist, and geneticists/genetic counselors are available in 23, 26, and 19 sites respectively. Physical therapists are available in all sites. Respiratory care for adult dystrophic patients is available in 27 sites.

### Clinical trial experience

Phase I, II, III, and IV clinical trials were experienced in 5 (17 %), 13 (43 %), 23 (77 %), and 15 (50 %) sites, respectively. Four sites have engaged in sponsor-initiated or investigator-initiated clinical trials of DMD. Clinical research coordinators (CRC) are available in 21 sites (data not shown).

### Patient recruitment

We operated two investigator-initiated clinical trials using Remudy for recruitment: “Exploratory Phase Trial of NS-065/NCNP-01 in Duchenne muscular dystrophy” (NCT02081625) and “Phase II Study of NPC-14 (Arbekacin Sulfate) to Explore Safety, Tolerability, and Efficacy in Duchenne Muscular Dystrophy” (NCT01918384).

### Inquiries and support

#### Feasibility inquiry

One pharmaceutical company requested the coordinating office to conduct feasibility inquiries regarding their clinical trials. The MDCTN completed those inquiries within 4 weeks.

### Meeting and education

Meeting opportunities, such as seminars and workshops, are generally welcome, since these are valuable opportunities to exchange information with other healthcare providers who are involved in neuromuscular disease treatment, as well as pharmaceutical companies that have developed cutting-edge drugs and devices for the management of specific diseases, like DMD. Seminars for PTs and CRCs are also great opportunities to engage in discussions and communicate different experiences regarding the treatment and care of patients with neuromuscular diseases. These seminars are also effective for standardization of evaluations.

## Discussion

Therapeutic agents for the treatment of rare disease are called “orphan drugs” and have become the object of attention of researchers and pharmaceutical companies in recent years. In 2012, the MHLW announced the 5-year Clinical Trial Activation Plan for the purpose of stimulating clinical research and trials in Japan. As part of that plan, relevant aims were creating a system to operate the clinical trials and establishing a patient registry of rare diseases [9]. To achieve these objectives effectively and to promote clinical trials and clinical research, collaboration was established between the patient registry and MDCTN. A close collaboration with the patients’ registry and standardization of treatment and clinical evaluations among many local sites is very important. It helps increase the availability and accessibility to the latest treatment information and increases the participation of patients with rare diseases and their families in clinical research, regardless of their area of residence. The Rare Diseases Clinical Research Network, supported by the National Institutes of Health (NIH), stated that the success of recruitment depends on many approaches, such as contact registries, referrals from patient advocacy groups, and enrollment of existing patients at consortium sites [10]. The MDCTN is the first national network aimed at facilitating regular and successful operations of clinical trials and research of neuromuscular diseases that comprises medical sites with a long-standing history of treatment for neuromuscular diseases in Asia.

When comparing our registered data to that in TREAT-NMD CTSR, the data for the proportion of patients with dystrophinopathy was similar. The ratio of LGMD is about two-thirds of that registered in TREAT-NMD. The proportions of DM1 and congenital muscular dystrophy (CMD) are 23 % and 7 % (Fukuyama type congenital muscular dystrophy: FCMD 5 %, other CMD 2 %) respectively, which is higher than the ratio in TREAT-NMD (17 % and 9 %, respectively) [11]. There is a remarkably higher proportion of patients with FCMD registered in MDCTN in than in TREAT-NMD CTSR. FCMD is a characteristic neuromuscular disease in the Japanese and Asian populations [12].

Regarding the cooperative sites, over 70 % of the sites can provide general and muscular evaluations. These data suggest that they have enough potential to conduct clinical trials. However, the MDCTN has two limitations regarding the proportion of patients with each disease: the accuracy of the numbers reported and quality of diagnoses. First, the proportion of each patient affected by each neuromuscular disease might be overestimated, especially in large urban areas. Because of the proximity of several sites, the registration of some patients might be duplicated in different sites and reports. Second, not all of the patients were genetically diagnosed because genetically diagnostic techniques were not available at the time of their diagnosis. However, registry data of MDCTN sites are still valuable for the conduction of clinical trials because there are no previous data on the infrastructural situation of clinical sites in Japan, such as that of TREAT-NMD.

There are both differences and similarities between MDCTN and other disease-specific networks, such as CINRG and TREAT-NMD. The main differences between MDCTN and the international networks are that MDCTN is a domestic network, and not all its sites have vast clinical research experience. Its main similarity with CINRG is that the coordinating office works as an ARO. The main similarities with TREAT-NMD are that both apply corroboration with patient registries, standardization of treatment, and establish outcome measures. The MDCTN is equipped with these functions, which are modified depending on the situation in Japan and are going to be improved.

In Japan, the MDCTN has conducted two patient recruitments for clinical trials, as mentioned above. Additionally, we have conducted one feasibility enquiry. Basically, consent for research is based on the relationship between the physicians, the patients, and their family [13]. Before the potential participants join the clinical trial through the recruitment process via the patient registry, we have arranged for patients to be introduced to the site by their treating physicians, with whom trust has been already established. We believe that it is very difficult to recruit the potential candidates through the system.

MDCTN has three advantages. The first one refers to quick inquiries and patient recruitment. To achieve quick inquiries and patient recruitment, a close communication with the patient registry and having updated registry data from all the sites are remarkably important. These data will indicate the location of the patients, which is useful for clinical research because it allows them to easily recognize potential research partners. This can also translate into less time and costs of recruitment for pharmaceutical companies. Additionally, showing the proportions of patient with rare diseases and sites may create opportunities for the Japanese sites to participate in international clinical research.

The second advantage is to be able to share the information of the latest research and clinical trials through the meetings. The MDCTN covers most of the hospitals in Japan with extensive experience in treating neuromuscular diseases. This makes it possible to provide equal opportunities in terms of the latest research information for the benefit of the patients.

There is no doubt that the support of a national network in collaboration with a national patient registry is a powerful source of support for an international network, such as TREAT-NMD and CINRG, by which clinical research for neuromuscular diseases is being promoted energetically. The conduction of global clinical trials is also very important for the development of cutting-edge drugs and treatments, especially for rare diseases. However, cooperative sites are not equally participative all over the world. It may be the case that only a few sites attend the activities from some countries in Asia. This may result in those sites gaining updated international, high-quality evaluation method, research information and/or treatment, and the rest of the sites may be left behind. MDCTN provides resources such as meetings and educational opportunities for health care providers, including PT and CRC. Furthermore, this is a good opportunity to share the latest information, treatment or evaluation for specific rare diseases. We believe that this will contribute to standardization among cooperating sites.

Ando et al. are reported that 15 % of multinational clinical trials were operated in Asia or East Asia in 2012 [14]. In this area, the Asia Oceania Myology Center (AOMC) provides opportunities for standardization of treatment and basic research. The AOMC also provides a forum for the discussion of issues related to their own countries, as well as support to find solutions. Therefore, it is clear that networks operating at a nation level, like MDCTN, are necessary. Each country has different issues that affect the conduction of clinical trials, such as medical environment, drug approval processes and regulations, and social systems, and thus, each country needs to develop different plans and strategies to tackle these issues. The MDCTN can serves as a model of a national network. Additionally, we assure that the activity of improving the national standards will contribute to improve and facilitate international clinical research activities.

## Conclusion

The MDCTN was established for the purpose of facilitating “the development of models of pharmaceuticals and medical devices for rare diseases at a national level” and to become an infrastructure for the support of multi-institutional clinical research of promising drugs, devices and health care modalities for the management of rare disease, such as neuromuscular dystrophy. Sharing updated health care information and standardized evaluations among cooperative sites makes it possible to activate qualified clinical research even if neuromuscular dystrophy is a rare disease. A close collaboration with the patient registry is important for recruitment of patients with rare diseases. We consider that network activities at a national level will contribute to successful international clinical research with high quality outcomes and improvement of patient care and treatment.

## Abbreviations

AOMC, Asia Oceania Myology Center; ARO, Academic Research Organization; BMD, Becker muscular dystrophy; CINRG, Cooperative International Neuromuscular Research Group; CRC, Clinical Research Coordinator; CT, Compute Tomography; CTSR, Care and Treatment Site Registry; DM, Myotonic Dystrophy; DMD, Duchenne Mascular Dystrophy; ECG, electrocardiogram; FCMD, Fukuyama type neuromuscular dystrophy; GNE, UDP-N-acetylglucosamine-2-epimerase/N-acetylmannosamine kinase; IRB, Institutional Review Board; MDCTN, muscular dystrophy clinical trial network; MHLW, Ministry of Health, Labour and Welfare; MLPA, multiplex ligation-dependent probe amplification; MRI, magnetic resonance imaging; NCNP, National Center of Neurology and Psychiatry; NHO, National Hospital Organization; PCR, polymerase chain reaction; PT, Physical Therapist; Remudy, Registry of Muscular Dystrophy; SMA, Spinal muscular dystrophy; TMC, Translational Medical Center.
